# F38. WINTER-LIKE PHOTOPERIOD GESTATION DELETERIOUSLY AFFECT EXPERIENTIAL- BUT NOT EXPRESSIVE-RELATED BEHAVIORS, WHILE ADULT PHENCYCLIDINE TREATMENT INCREASES EXPERIENTIAL BEHAVIORS: RELEVANCE TO SCHIZOPHRENIA

**DOI:** 10.1093/schbul/sby017.569

**Published:** 2018-04-01

**Authors:** Molly Kwiatkowski, Zackary Cope, Chuck van de Chapelle, Davide Dulcis, Susan Powell, Jared Young

**Affiliations:** University of California, San Diego

## Abstract

**Background:**

Patients with schizophrenia exhibit negative symptoms that predict functional outcome. Understanding the mechanisms underlying these symptoms are important for developing novel therapeutic treatments. These negative symptoms can be grouped into experiential- (motivational) or expressive- (social) based behaviors testable in rodents. Increased incidences of schizophrenia have been reported with winter gestation, while antagonizing NMDA receptor function in adulthood has been used to model other symptoms of schizophrenia. These two approaches were used to determine mechanisms that may contribute to negative symptoms in schizophrenia and test potential therapeutics.

**Methods:**

Normal dam mice were housed in custom photoperiod chambers for one week under normal photoperiod conditions (12 hour light:12 hour dark) to allow for initial acclimation. Half of the photoperiod chambers were then shifted to a short active (SA) condition (19 hours light: 5 hours dark) with mating triads formed. After a two-week pairing, pregnant dams were single-housed. The resulting pups were reared under these photoperiod conditions until P28, at which time they were weaned by sex into tetrads and moved into a standard vivarium room under normal active (NA) photoperiod. Mice were trained to perform nose-poke responses in a five-choice operant chamber. Adult Long Evans rats were also trained in separate 5-choice chambers and repeatedly treated with phencyclidine (PCP). Motivational behavior was tested on a progressive ratio breakpoint (PRB). Social behavior of mice were also assessed using a 3-chocie social recognition paradigm.

**Results:**

In PRB, breakpoint was decreased in WT mice reared in SA vs. NA photoperiod (F(1,66)=4.4, p<0.05). In contrast however, no deficits in social interaction was observed (F<1, ns). Unlike the SA gestation-induced reduction in breakpoint, subchronic PCP increased breakpoint 1 [t(21)=5.3, p<0.0001], 7 [t(21)=2.2, p<0.0001] and 14 days after treatment to rats.

**Discussion:**

Winter-like photoperiod births in mice induced psychiatry-relevant amotivation as measured by breakpoint, though did not affect social interaction or recognition. In contrast, subchronic PCP treatment increased breakpoint in rats tested 1 and 7 days after treatment. Hence, neurodevelopmental mechanisms underlying the winter-like gestation likely contribute to experiential- but not expressive-related behaviors, while altered NMDA receptor function are unlikely to contribute to such experiential-related abnormalities. Ongoing work will characterize epigenetic, synaptic, and/or system-level adaptations underlying developmental differences between NA and SA photoperiod born mice as well as defining critical periods throughout gestation and rearing that are driving these effects.

